# Editorial: Endotheliopathies: Current concepts and importance in clinical practice

**DOI:** 10.3389/fmed.2023.1162121

**Published:** 2023-03-03

**Authors:** Eleni Gavriilaki, Panagiota Anyfanti

**Affiliations:** ^1^Second Propedeutic Department of Internal Medicine, Aristotle University of Thessaloniki, Thessaloniki, Greece; ^2^Second Medical Department, Aristotle University of Thessaloniki, Thessaloniki, Greece

**Keywords:** endotheliopathies, endothelial dysfunction, endothelial injury syndromes, COVID-19, hematological disorders, cardiovascular disorders, hematopoietic cell transplantations

Over the last years, our understanding of endothelium has greatly evolved (1, 2) (Anyfanti et al.). Cardiovascular and hematological disorders, as well as hematopoietic cell transplantation, are considered key fields in which endothelial dysfunction has been studied. The list of endothelial injury syndromes is constantly updated, including not only toxicity syndromes but also novel entities, such as the coronavirus disease-19 (COVID-19) (3–5).

Despite the plethora of studies, the clinical significance of endothelial dysfunction remains under investigation. Better understanding of current concepts and significance in clinical practice emerges as extremely important to set the ground for the development of therapeutic approaches specifically targeting the endothelium. Several questions remain unanswered in this complex field.

This Research Topic gathered Original Research, Brief Research Report, and Mini Review articles, focusing on endothelial dysfunction or endothelial injury studies in the following areas:

Novel entities recognized as endotheliopathies, such as COVID-19
Cardiovascular disorders
Hematological disorders
Hematopoietic cell transplantation
Chronic inflammatory disorders

All articles submitted to us for this Research Topic underwent a rigorous peer review process. Ultimately, eleven articles were published.

(i) In pre-eclamptic patients, phosphatidylserine exposing extracellular vesicles were increased and associated with global hemostatic parameters and fibrin clot properties (Lalic-Cosic et al.).

(ii) In systemic sclerosis, up-to-date knowledge of cellular and molecular aspects in vasculopathy, as well as therapeutic approaches were reviewed (Zanin-Silva et al.).

(iii) In essential hypertension, pathophysiological evidence of endothelial dysfunction in cardiovascular diseases and potential innovative therapeutic strategies were reviewed (Gallo et al.).

(iv) In pulmonary essential hypertension, vascular remodeling and its potential involvement of innate and adaptive immunity were reviewed (Tobal et al.).

(v) In systemic sclerosis, uric acid was significantly associated with the capillaroscopic patterns, reflecting a progressive microvasculopathy (Pagkopoulou et al.).

(vi) In the life-threatening field of thrombotic microangiopathies, complement-mediated damage was reviewed (Blacso et al.).

(vii) In COVID-19, hematological abnormalities were associated with type I interferon pathway activation and disease outcomes (Georgakopoulou et al.).

(viii) In Takayasu artiriitis, cardiovascular risk directly associated with diastolic dysfunction and inflammatory cell infiltration in the vessel wall (Cicco et al.).

(ix) In psoriasis, circulating and vascular biomarkers of endothelial dysfunction were summarized, and the impact of systemic psoriasis treatments on endothelial dysfunction and patients' cardiovascular risk was discussed (Anyfanti et al.).

(x) In secondary thrombotic microangiopathies, loss of glycocalyx integrity impaired complement factor H binding and cyclosporine-induced endothelial cell injury (Teoh et al.).

(xi) In thrombotic thrombocytopenic purpura, the PLASMIC score was applied in risk prediction of a real-world cohort (Lee et al.).

Taking into account the multi-disciplinary character of this Research Topic, we hope that it will inspire researchers to continue their explorations into novel advances in their fields.

## Author contributions

All authors listed have made a substantial, direct, and intellectual contribution to the work and approved it for publication.
